# Addressing antimicrobial resistance through community engagement: a framework for developing contextually relevant and impactful behaviour change interventions

**DOI:** 10.1093/jacamr/dlad124

**Published:** 2023-11-24

**Authors:** Jessica Mitchell, Helen Hawkings, Sophia Latham, Fariza Fieroze, Abriti Arjyal, Dani Jennifer Barrington, Sushil Baral, Md Badruddin Saify, Paul Cooke, Prudence Hamade, Rumana Huque, Ayuska Parajuli, Amam Zonaed Siddiki, Rebecca King

**Affiliations:** Nuffield Centre for International Health and Development, Leeds Institute for Health Sciences, Faculty of Medicine and Health, University of Leeds, Woodhouse, Leeds LS2 9JT, UK; Malaria Consortium, The Green House 244-254 Cambridge Heath Rd, London EC2 9DA, UK; Department of Livestock and One Health, Institute of Veterinary and Ecological Sciences, University of Liverpool, Leahurst Campus, Chester High Road, Neston CH64 7TE, UK; ARK Foundation, Suite C3 & C4, House 06, Road 109, Gulshan-2, Dhaka 1212, Bangladesh; HERD International, Bhaisepati, Lalitpur, Nepal; School of Population and Global Health, The University of Western Australia, 35 Stirling Highway, Crawley 6009, Western Australia; HERD International, Bhaisepati, Lalitpur, Nepal; ARK Foundation, Suite C3 & C4, House 06, Road 109, Gulshan-2, Dhaka 1212, Bangladesh; Faculty of Arts and Humanities, Centre for World Cinemas and Digital Cultures, University of Leeds, Woodhouse, Leeds LS2 9JT, UK; Malaria Consortium, The Green House 244-254 Cambridge Heath Rd, London EC2 9DA, UK; ARK Foundation, Suite C3 & C4, House 06, Road 109, Gulshan-2, Dhaka 1212, Bangladesh; HERD International, Bhaisepati, Lalitpur, Nepal; Faculty of Veterinary Medicine, Chattogram Veterinary and Animal Sciences University, Chattogram 4225, Bangladesh; Nuffield Centre for International Health and Development, Leeds Institute for Health Sciences, Faculty of Medicine and Health, University of Leeds, Woodhouse, Leeds LS2 9JT, UK

## Abstract

**Background:**

Community engagement (CE) interventions often explore and promote behaviour change around a specific challenge. Suggestions for behaviour change should be co-produced in partnership with the community. To facilitate this, it is essential that the intervention includes key content that unpacks the challenge of interest via multiple sources of knowledge. However, where community lived experience and academic evidence appear misaligned, tensions can appear within the co-production dynamic of CE. This is specifically so within the context of antimicrobial resistance (AMR) where ideal behaviours are often superseded by what is practical or possible in a particular community context.

**Methods:**

Here we describe a framework for the equitable development of contextually appropriate, clearly evidenced behavioural objectives for CE interventions. This framework explores different sources of knowledge on AMR, including the potentially competing views of different stakeholders.

**Findings:**

The framework allows key content on AMR to be selected based upon academic evidence, contextual appropriateness and fit to the chosen CE approach. A case study of the framework in action exemplifies how the framework is applicable to a range of contexts, CE approaches and One Health topics beyond just AMR.

**Conclusions:**

Within CE interventions, academic evidence is crucial to develop well-informed key content. However, this formative work should also involve community members, ensuring that their contextual knowledge is valued. The type of CE approach also needs careful consideration because methodological constraints may limit the breadth and depth of information that can be delivered within an intervention, and thus the scope of key content.

## Introduction

Community engagement (CE) is a popular approach for addressing complex challenges ranging from migration to climate change and public health.^1–5^ Over the past decade, CE has increasingly been used as part of the One Health approach, which addresses issues impacting upon human, animal and environmental health.^2–4,6–9^ There are many definitions of CE (Table 1), but it is commonly referred to as a *process* of engagement that develops trust and partnerships between a specific group of people (community) and wider stakeholders (such as policymakers, service providers and research teams). Through Ce, these teams work together to develop contextually appropriate interventions that address a given challenge. This can happen in multiple directions by enhancing internal communication to develop more effective community-led solutions, but also by supporting communities to inform policy-level discussion and facilitate interactions with stakeholders they may not usually have access to. Often CE interventions will facilitate an exchange of knowledge and experiences, which lead to an exploration of the challenge, development of appropriate solutions and ultimately facilitate changes in behaviour.^2,5,10–13^ This is an intentional process of equitable co-production where the exact routes to behaviour change will not be known or prescribed by the research team but rather co-developed with the community itself through the course of the intervention.^1,5,9,14^ To achieve behaviour change, CE interventions are guided by *key content* rather than *key messages.* This terminological distinction is important. *Key messages* are expected within educational or awareness-raising materials, which aim to deliver specific information on a topic. However, within CE approaches, although these key messages are just as relevant, the process of engagement is very different. In Ce, *key content* aims to distil information about the focal topic but allow room for community interpretation.^2,6,15,16^ However, it can be difficult to formulate content in a way that allows both community involvement *and* acknowledges the academic evidence around the challenge.^2,16,17^

**Table 1.** Definitions of CE and their source material

Organization	Definition of CE	Link
Community Engagement for Antimicrobial Resistance (CE4AMR)	A participatory process through which equitable partnerships are developed with community stakeholders, who are enabled to identify, develop and implement community-led sustainable solutions using existing or available resources to issues that are of concern to them and to the wider global community.	https://ce4amr.leeds.ac.uk/about/
United National’s Children’s Fund (UNICEF)	A foundational action for working with traditional, community, civil society, government, and opinion groups and leaders; and expanding collective or group roles in addressing the issues that affect their lives. Community engagement empowers social groups and social networks, builds upon local strengths and capacities, and improves local participation, ownership, adaptation and communication. Through community engagement principles and strategies, all stakeholders gain access to processes for assessing, analysing, planning, leading, implementing, monitoring and evaluating actions, programmes and policies that will promote survival, development, protection and participation.	https://www.unicef.org/mena/reports/community-engagement-standards
WHO	A process of developing relationships that enable stakeholders to work together to address health-related issues and promote well-being to achieve positive health impact and outcomes.	https://www.who.int/publications/i/item/9789240010529
FAO	Risk communication and community engagement (RCCE) refers to the processes and approaches to systematically consult, engage and communicate with people and communities who are at risk, or whose practices or behaviour affect risk. The aim is to encourage, enable and include stakeholders in the prevention of and response to risks by adapting communication to local political, economic, social, cultural, psychological and other realities. In the case of COVID-19, RCCE enables authorities and communities to work together to promote healthy behaviour and reduce the risk of spreading infectious diseases.	https://www.fao.org/3/cb0526en/CB0526EN.pdf
National Institute for Health and Care Research (NIHR)	NIHR uses the term community engagement intervention (CEI) to refer to patient and public involvement (PPI), which is used within the UK, in the global health context. The two terms are both used to describe how members of the public’s voices can be heard in research, and both are underpinned with the commitment to promote inclusion.	https://www.nihr.ac.uk/documents/nihrs-vision-and-goals-for-community-engagement-and-involvement-in-global-health-research/28271#:~:text=NIHR's%20community%20engagement%20and%20involvement,affected%20by%20the%20research%20outcomes

Research teams often consolidate expertise around the focal challenge to provide evidence upon which to build intervention content. Systematic and scoping reviews are useful approaches for identifying evidenced-based topics^18–20^ whilst Delphi-style exercises can develop content based on perceived importance of ‘expert’ groups.^21,22^ However, despite providing valuable empirical support, such methods can make assumptions about what messages are contextually relevant.^23,24^ This in turn may prevent the community from sharing their own knowledge and experiences within the intervention. For example, the academic literature suggests that over-the-counter purchasing of medical and veterinary antimicrobials is a key behaviour driving drug resistance across the world.^25–31^ Based on such a finding it may be simple to assume that a key message for any CE and antimicrobial resistance (AMR) intervention would be ‘*do not buy antimicrobials over the counter, instead source a prescription*’. However, in many contexts this may not be possible. Rural, low-resource communities may not be able to access a prescription provider,^30,32^ whilst other communities may not be comfortable trusting and navigating formal healthcare systems.^32^ Hence, there are many layers of complexity around the reasons how and why people source antimicrobials; a review or Delphi-based methods may not accurately capture these nuances. Alternatively, projects may take a co-production approach to content development, allowing communities to choose what information is shared and what an appropriate delivery mechanism may look like.^2,9,10,14,33^ Here community-generated data can inform content and allow the development of contextually relevant behavioural objectives. This gives much greater ownership and so results in more effective, community-based interventions.^17,34^ Co-production approaches can also help to gauge community-level buy-in to a topic and to discover colloquial terminology and stories, which can help contextualize the problem in relevant ways for a given community.^35^

A major challenge with co-production of *key content* is ensuring that information is factually accurate, evidence-based and representative of the experience of the focal community. Multiple sources of knowledge including community generated perspectives and academic evidence must be synthesized, and this process can degrade community trust if they feel their information is being ‘checked’ or ‘corrected’.^9,34,36^ Synthesizing such different forms of knowledge and evidence can take an iterative approach, engaging with different stakeholders and sources of evidence at different timepoints. A concern here is that the knowledge shaping the project’s design and content will be more closely rooted in academic evidence and the input of the community comes later.^9,37^ Hence, at the point of content development, finalizing behavioural objectives for CE interventions can be fraught with equity and integrity concerns. Content must also be appropriate to the type of intervention being conducted.^16^ For example, regular meetings between the same community members may allow a challenge to be explored in more detail and so facilitate the inclusion of more complex behavioural objectives, whereas events designed to engage new attendees each time may require headline content, which can be repeated many times. Again, this process of fine-tuning content to modes of delivery gives the opportunity for knowledge providers to feel overlooked if their contribution is omitted.

Evidence-based and contextually appropriate content is essential to create CE experiences that are relevant to the focal community and accurately distil information. However, a balance needs to be struck between co-production of material that reflects the lived experience of the focal community alongside robustly evidenced facts that promote health and do not cause harm.^34,36^ This publication describes a framework for developing *key content* for CE interventions that are evidence-based and contextually and methodologically appropriate. Through case studies we apply the framework to two iterations of a community dialogue approach (CDA) to tackle AMR in two difference contexts, and in doing so consider the nuances of content co-creation for CE interventions.

### Objectives

The objectives of our study were to: (i) describe a robust framework for developing *key content* for CE interventions that are both evidence-based and contextually and methodologically appropriate; (ii) apply this framework to create behaviour-change objectives for a CDA to tackle AMR; and (iii) reflect on the challenges around creating *key content* for CE interventions.

## Materials and methods

We developed a 10-step framework for creating *key content* in CE interventions (Figure 2) as part of the COSTAR project (**CO**mmunity-led **S**olutions **T**o **A**ntimicrobial **R**esistance).

### COSTAR project: context

COSTAR seeks to develop and robustly evaluate the ability of the CDA to tackle the One Health challenge of AMR. AMR refers to the way microbes, including bacteria, change to resist treatments such as antibiotics and pesticides. AMR caused 1.27 million human deaths in 2019, with this figure predicted to rise to 10 million annually by 2050.^38^ AMR is also predicted to lead to a significant reduction in livestock productivity as food-producing animals succumb to drug-resistant infections.^39^ AMR is a natural process but is accelerated by behaviours such as taking antimicrobial treatments for the wrong amount of time, when they are not needed, or when they are not matched to specific infections.^26,27,29,40,41^ The latter often occurs when purchased over the counter rather than by prescription from a qualified healthcare provider or vet.^26^ Thus, AMR is considered a social and behavioural challenge, which is seemingly suited to exploration via CE.^8,42–44^

**Figure 1.**
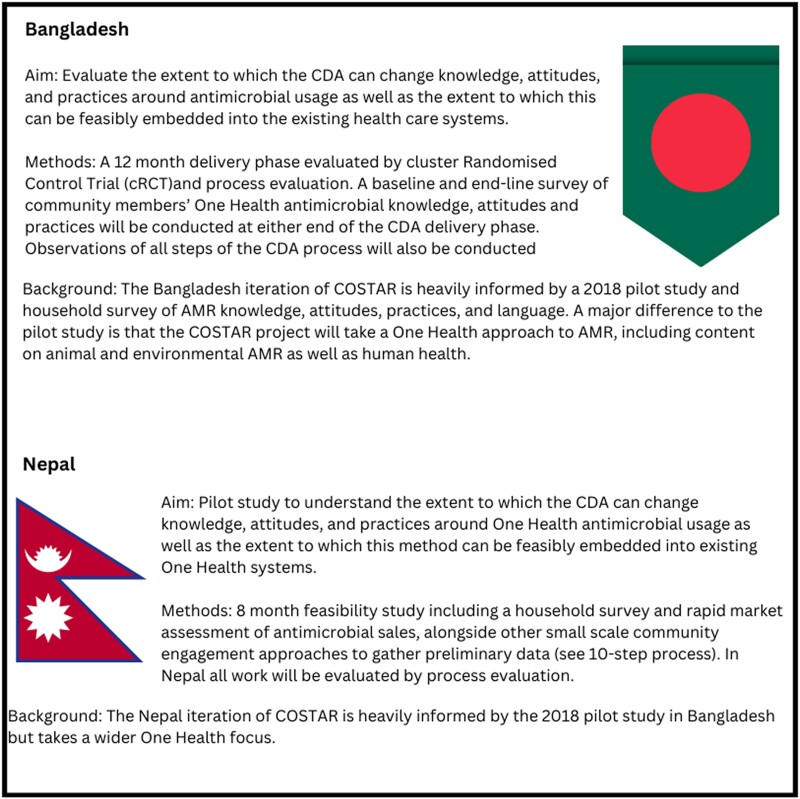
A summary of key differences between the COSTAR interventions in each country (Bangladesh and Nepal).

The CDA is a recognized CE method for which a full description and implementation guide have been published.^45^ In brief, the CDA involves training community volunteers on a specific challenge, and on facilitation techniques. These people then act as facilitators and host regular dialogues within their communities to discuss issues around the given challenge (in this case AMR). Community members explore the challenge, identify solutions and plan for action at community level. They have been successful in a range of contexts across Africa, including the management of malaria, pneumonia, diarrhoea and neglected tropical diseases.^45–48^ In 2018, the COSTAR team piloted a 6 month CDA intervention to address antibiotic resistance in human health within Bangladesh.^10,49^ The team is now developing this intervention further to include all forms of antimicrobials and the broader One Health content in both Nepal and Bangladesh, while noting that there are key differences in each setting. Alongside CDA, the COSTAR team also used a variety of small CE interventions to ensure local knowledge was being exchanged through the process of development (see Figure 1 and Case study, available as Supplementary data at *JAC-AMR* Online).

### The 10-step framework

**Figure 2.**
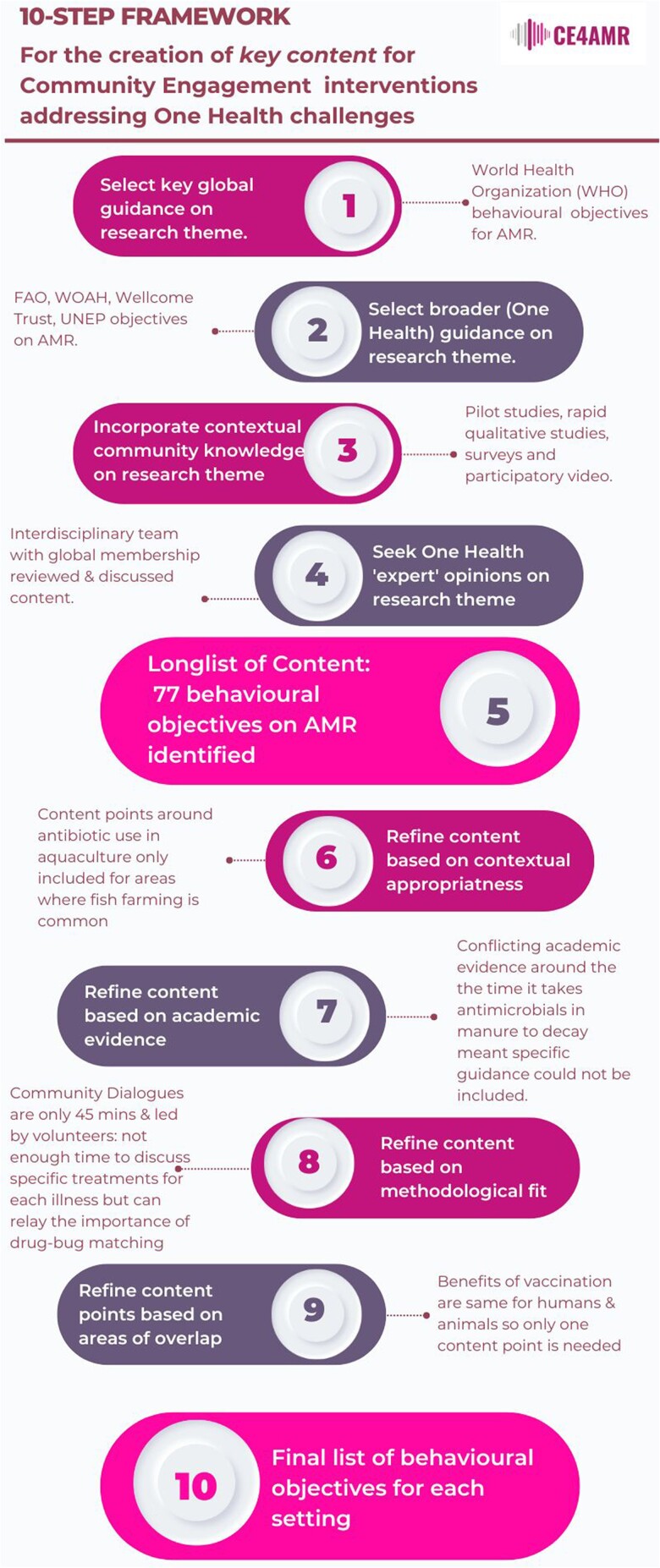
The 10-step framework for the selection of *key content* points for CE interventions that address complex One Health challenges, using the worked example of AMR.

A crucial initial phase within the COSTAR project was to develop simple behavioural objectives around AMR that were applicable to community-level actors, and which could be used to guide content and material development for the CDA intervention. It was through this process of amalgamating academic, community, contextual and methodological knowledge that the team developed a 10-step framework for the creation of contextually appropriate but well-evidenced *key content* to guide CE interventions (Figure 2).

The first five steps draw together multiple sources of knowledge around the focal research theme. Step 1 begins with global guidance on the research theme, in our case the WHO behavioural objectives on AMR. These objectives acted as the starting point for the COSTAR content development process, having the weight of WHO expertise and global recognition behind them. For challenges other than AMR it would of course be at the discretion of the research team to decide what their focal global guidance would be and how to justify this. Ideally this would be the overarching, broad guidance created by a world-leading authority on the topic such as the WHO. Selecting a single piece of global guidance as a starting point allows *key content* development to begin from a reputable, well-evidenced starting point. In step 2, the framework then asks what broader guidance can be incorporated to better understand the challenge in question; in the case of the COSTAR project this included AMR guidance from the Food and Agriculture Organization (FAO), Wellcome Trust and the World Organisation for Animal Health (WOAH) (see Table S1, available as Supplementary data at *JAC-AMR* Online) for lists of all documents considered). Again, for other challenges a similar selection approach could be taken by asking: beyond your key piece of global guidance, what other actors provide information on this topic and can you find the globally recognized documentation that represents all One Health aspects of this challenge? Step 3 then considers contextual information from the field site and local community. For COSTAR this was gathered from pilot work, including surveys, rapid qualitative studies and participatory interventions such as participatory video (see Table S2 for more detail). In step 4, the wider expertise of stakeholders within the research theme are used to assess the content points and suggest areas for expansion or consolidation; in COSTAR this was achieved by interactive online discussions using breakout rooms in software such as Zoom and Microsoft Teams. Step 5 is a reflection point for the research team to assess the knowledge amassed and consider if other input is needed (Table 2). From steps 6–9 the framework seeks to refine this information into distinct behavioural objectives by applying a series of lenses, namely step 6: contextual appropriateness, Step 7: academic rigour, and step 8: methodological fit. These steps will look different depending on the One Health challenge of focus and the method of CE utilized but we provide detailed examples in our case study. Step 9 considers where areas of overlap in content could be addressed and condensed. Finally, step 10 is another reflection point whereby the research team may consider the need to repeat certain steps or bring in new sources of knowledge if necessary (Tables 3 and 4).

**Table 2.** The 77 *key content* points developed from a review of global guidance on AMR (Table S1) and a variety of CE activities with stakeholders in Bangladesh and Nepal (see Table S2 for more detail on CE activities)

Proposed content point	
General AMR	
1	Common infections that were easily treatable are becoming more dangerous and killing once again because of drug-resistant infections, which can no longer be cured by modern medicine.
2	This is happening because microbes are finding ways to fight off our medicines; we say they are becoming resistant to medicines. This is called AMR.
3	AMR is happening across the world and is increasing.
4	One of the reasons AMR happens is because we do not use our antimicrobial medicines properly.
5	When a person or animal catches a drug-resistant infection, they can be sicker and less productive for longer, it may cost more to treat the illness, they could even die.
6	If AMR continues to happen many people and animals will get very sick and die.
7	One of the reasons AMR happens is because we do not use our antimicrobial medicines properly.
Key background knowledge points	
9	Different infections cause different diseases, which require different types of treatment.
10	These different diseases may have similar symptoms; the only way you can tell what infection or disease you have is by going to a qualified healthcare professional.
11	Your healthcare professional will tell you what medicine or treatment you need; you should follow their advice.
12	The healthcare professional’s advice may be to take an antimicrobial medicine, but it may be another type of medicine.
13	Ask your healthcare provider what medicine you are supposed to take, for how long, and why.
14	If, after following advice you/your child/your animal does not get better, go back to your healthcare professional for more advice.
15	Antimicrobials are medicines that save lives by treating infections cause by microbes.
Human health	
16	To find out what type of medicine you might need, seek advice and treatment from a qualified healthcare provider.
17	The symptoms of many illnesses can look the same (i.e. fever, lethargic etc.) but they may be caused by different infections. Health professionals may conduct tests to find out which infection is making you/your animal ill and then prescribe the right medicine.
18	Do not take old medicine you have at home just because it worked last time.
19	Antimicrobials are one type of treatment/medicine you may be given.
20	Antimicrobials are live-saving medicines, which must be protected.
21	Using antimicrobials properly saves lives.
22	Using antimicrobials properly.
23	Buying and completing the dose/course.
24	Only buying antimicrobials when you have a prescription for them.
25	Not demanding antimicrobial medicines if your healthcare provider says you don’t need them.
26	Be patient with your healthcare provider if they ask you to take a diagnostic test.
27	Do not share, save or store leftover antimicrobials.
28	Do not be tempted to use the same medicine you/your child/animal had last time you were ill; go back to your healthcare professional for advice.
29	If you don’t get better, go back to the healthcare professional for more advice.
30	Do not use antimicrobials as growth promotors.
Linking health to AMR	
31	Using the wrong medicine, in the wrong amount or for the wrong amount of time could cause AMR.
32	This is because the microbe causing your illness will learn how to fight off the medicine.
33	Take the right medicine for the right infection and get help from a professional to make this decision. Conversely, it is important to use antibiotics at the right time for the right duration. This will ensure they remain effective in the future.
34	Antimicrobials can come in liquid or tablet form; the liquids can be injected, in a drip or ingested.
35	No method of antimicrobial delivery is better than the other. Your healthcare professional will advise on the method you/your family/your animal need for your illness; do not demand a specific type.
36	Expensive antimicrobials are not always better or stronger than cheaper antimicrobials.
Preventing infection	
37	Infection prevention is a good way to stay healthy; this will keep you and your animals productive and minimize the amount you have to spend on medicines. It will also minimize the likelihood of using antimicrobials.
38	Handwashing with soap or alternative at key times (after contact with faeces and before eating or preparing food/breastfeeding) or, at the very least, after using the toilet.
39	Keeping food preparation areas clean for humans and animals.
40	Cleaning homes and animal shelters regularly.
41	Cleaning animal feed and water stations.
42	Keeping sick humans and animals away from healthy humans and animals until they get better.
43	Eating fresh, clean, cooked nutritious food.
44	Not sharing used water between humans, animals, and fish.
45	Keeping human and animal vaccinations up to date.
Animal health	
46	AMR can also happen in the microbes that cause animal infections.
47	We also need to be careful how we use medicines in animal health because if we use the wrong medicine the animal could be sicker for longer, less productive and cost a lot to treat.
48	Consult a veterinarian before administering antimicrobial drugs to livestock or domestic animals.
49	Regular veterinary visits will ensure your livestock and domestic animals remain healthy.
50	Keep your livestock and domestic animals up to date with their vaccinations.
51	Do not save leftover antimicrobials for later use.
52	Return any leftover antimicrobials to agro vet if possible.
53	Do not use antimicrobials prescribed for animals to treat humans (or vice versa).
54	Do not use antimicrobials as growth promotors.
55	Avoid consuming the meat, milk, eggs of animals who are sick or on antimicrobial treatments.
Aquaculture	
56	Cover fish-farm ponds to prevent wild animals feeding on the farmed fish.
57	Do not add antimicrobials to fishponds or fish food.
58	Seek veterinary advice on how to treat sick fish.
59	Clean fishponds regularly.
60	Do not use the water from fish farm ponds for washing, drinking, cleaning or to provide to animals.
61	Avoid consuming fish that are ill or have been treated by antimicrobials.
Water, sanitation and hygiene (WaSH)	
62	Resistant infections and antimicrobials can move around in water.
63	1. Wash and dry hands with clean water and soap or alcohol gel where possible.
	2. After working with livestock and domestic animals.
	3. After toileting.
	4. Before eating and food preparation.
	5. Before breastfeeding.
	6. After cleaning babies.
64	Clean water should be prioritized for drinking, plus hygiene-related activities such as washing the body, food and cooking utensils.
65	If you are unsure how clean your water is, boil it before use.
Environment	
66	Environment = the places and spaces where people, animals, plants, and microbes live.
67	Keeping the environment clean and healthy can keep people and animals clean and healthy.
68	Infections can live in soil and water.
69	Infections can live in human and animal waste.
70	Antimicrobial traces can be present in human and animal waste.
71	Store animal waste products away from water sources.
72	Do not defaecate into the open environment.
73	The rainfall and seasonality could affect where it is best to store manure.
74	Compost manure for around 8 weeks before using on your fields.
75	Tidy up leftover food sources from human and animal settings to avoid attracting wild animals.
76	Cover fishponds to avoid wild animals predating on the farmed fish.
77	Do not dump antimicrobial waste in the environment.

These 77 objectives reflect step 5 of the framework (Figure 2).

**Table 3.** The final *key content* points created for the Nepal iteration of the COSTAR project to create community-level action on AMR

Key message	Original content points considered (refer to Table 2)	Community dialogue session number							
		1	2	3	4	5	6	7	8
Different illnesses in people and animals are caused by different microbes.	1	X	X	X	X	X	X	X	X
Different medicines are needed for different illnesses.	2		X		X			X	
Antimicrobials are one type of group of medicines; they come in many forms.	11, 15, 17, 19, 20	X	X						
Antimicrobials are lifesaving/life-enhancing medicines, but they only work properly when matched to certain microbes/illnesses/infections.	11, 15, 20, 21, 31–36 inclusive		X		X		X	X	
Ask your healthcare provider or vet to explain about the medicine you are given and how to take it. This should include the duration, route and frequency of taking medicine and any expected mild side effects, which should not stop you taking it as advised.	11, 12, 13, 14, 15		X	X		X		X	
It is not easy to know if you need antimicrobials or which antimicrobials you or your animals need so always seek advice/medicine from qualified/registered health professional or vet.	11–30 inclusive	X	X	X	X	X	X	X	X
Going to the health centres/vet centres when you or your animals are sick helps you to: Identify your illness. Get the right medicine to treat your illness. Save money as you will buy the needed medicine. Get the right amount of medicine.	11–30 inclusive			X		X		X	X
Do not self-medicate yourself, your family, or animals. Only a ‘qualified’ health worker can correctly diagnose your illness and provide the right drug for treatment.	11–30 inclusive		X	X		X		X	X
Don’t believe that all expensive antimicrobials/stronger medicines will always be better or needed to cure you/your children/your animals’ illness.	11–30 inclusive			X					
Antimicrobials are lifesaving medicines, but they can only work when used correctly.	11–30 inclusive				X				X
Taking the right medicine for the wrong amount of time will not cure your illness but can lead to a problem called AMR. Completing the full course/dosage of antimicrobials is equally important as drug–bug matching.	11, 12, 13, 14, 15				X	X		X	
Microbes can learn to become stronger than the antimicrobial medicines (say/give example for each type of antimicrobials); this is a process called AMR and when it happens, antimicrobial medicines can no longer treat diseases.	1–8 inclusive, 31–36 inclusive				X			X	
Through AMR, microbes change (but not the human or animal). AMR is very dangerous because the medicines that used to cure infections are not able to cure them anymore.	2, 8, 31, 32, 33				X			X	
Simple infections become more serious. More drugs or different drugs might be required, which could be more expensive, and the treatment and hospital stays might be longer.	5–8				X			X	
Causes of AMR are inappropriate uses of antimicrobial medicines such as antibiotics. Inappropriate use means: Not using correct medicines to treat microbes (drug–bug matching). Not taking antimicrobial medicines correctly: * *Not completing the full course as recommended by your healthcare provider. * *No. of days to be taken. * *Frequency of medicine to be taken/day. Taking antimicrobial when not needed.	1–8 inclusive, 31–36 inclusive				X	X		X	
Problem of AMR will get worse over time if we continue to neglect. Hence, we need to take immediate action.	3, 6				X				
There are simple behaviours we can all take to minimize AMR.	37–44 inclusive				X	X	X	X	X
Be patient with the medicines you are prescribed as it may take a few days to feel better.	31–36 inclusive, 45–54 inclusive					X	X	X	
Storing and sharing antimicrobials with other people or animals can lead to AMR because the other person or animal may not need the same treatment.	44, 50, 51, 52					X	X	X	
By preventing infections in both humans and animals, we can prevent unnecessary use of antimicrobials.	37–44 inclusive, 61–65 inclusive						X	X	X
Keep your animals and children up to date with vaccinations.							X	X	
Good animal husbandry is key to minimizing infection and illness.	48–60 inclusive						X	X	X
Increase your cleanliness practices if a person or animal is sick.	42						X	X	
Always wash your hands with soap and water: Before cooking and eating food. Before and after milking your animals (cows, buffalo). After using toilet, cleaning toilets of your child. After touching or caring for animals.	63						X	X	
Always use clean drinking water for both human and animal consumption.	41, 44, 55, 59, 64, 65						X	X	
Do not defaecate in the open field.	72						X	X	
Only give antimicrobials to sick animals and fish.	47–52, 56, 57							X	X
Do not give antimicrobials to healthy animals.	47–52, 56, 57							X	X
Microbes can move between humans, animals, plants and water so we need to take care of our environment to prevent infections spreading.	37, 45, 61, 68, 69						X		X
Antimicrobial medicines do not fully break down in human and animal bodies and this means traces of these medicines can pass out of our bodies in our waste or in the food products of animals.	54, 60, 74								X
Do not throw waste antimicrobials into toilets, latrines, water sources, soil or fields; instead return to health posts, pharmacies, and veterinary clinics.	77								X
Do not use excreta of animals taking antimicrobial treatments as a fertilizer straightaway.	54, 60, 74						X	X	X
Preventing antimicrobials getting into our environment is an easy way to minimize the risk of AMR.	66–77 inclusive								X
Remember to store human and animal waste away from drinking water sources.	71, 73, 76						X	X	X

**Table 4.** The final *key content* points created for the Bangladesh iteration of the COSTAR project to create community-level action on AMR

Key message	Original content points considered (refer to Table 2)	Community dialogue session number									
		1	2	3	4	5	6	7	8	9	10
Different illnesses in people and animals are caused by different microbes.	1		X								
Different infections need different treatments. Treatments may include medicines like antibiotics.	2		X						X		
Antibiotics are a common medicine that are used to treat bacterial infections. They come in different forms such as pills, injection, liquid or creams for use outside the body. Health professionals will advise you which one to use.	11, 15, 17, 19, 20		X								
Antibiotics save lives of humans and animals and make them feel better when taken correctly.	11, 15, 20, 21, 31–36 inclusive		X			X	X		X		
Only use antibiotics when prescribed by a certified health professional. (Same message for humans, fish and animals).	11–30 inclusive		X	X			X		X		
The symptoms of many illnesses can look the same, but they can be caused by different infections (bacteria, viruses, parasites or fungi).	11–30 inclusive			X			X		X		
Buying antibiotics without consulting a health professional could mean you are given the wrong medicine for your illness, too much or too little of the medicine.	11–30 inclusive			X			X				
Only use antibiotics when prescribed by a registered health provider to ensure the proper treatment of the infection and complete recovery. This will ensure they remain effective in the future.	11–30 inclusive			X			X				
Health professionals can conduct tests to find out which infection is making you/your animal ill so that they can prescribe the right medicine.	25, 26			X							
If you need to purchase antibiotics, always take your registered health provider’s prescription, and buy a complete course.	16–28 inclusive			X			X				
Expensive antibiotics are not necessarily better.	36			X							
Always follow your health worker’s/vet’s advice on when and how to use antibiotics. If humans and animals are to get better from illnesses, they need to make sure the right medicines are taken for the right amount of time and in the right amount (dosage).	21–36 inclusive			X	X						
Never save antibiotics for later or share antibiotics with another person or animal, as this poses risks for you and others.	27, 44, 50, 51, 52				X				X		
There should be no antibiotic leftover, but if there are any, they should be taken to the community health clinic and handed over to the health providers for proper use/disposal.	27, 50, 51				X						
It is important not to use antibiotics prescribed for humans on animals and vice versa. This will ensure they remain effective in the future.	21–36 inclusive, 50–52				X						
Do not throw the leftover or expired antibiotics down the toilet or in the open environment as they may harm the good bacteria.	51, 77				X						
Bacteria can learn how to ‘resist’ the effect of antibiotics and other medicines. This is called AMR.	31–36 inclusive					X					
AMR is very dangerous because infections that were easily treatable are killing once again because they have become drug-resistant infections, which means they can no longer be cured by modern medicine.	2, 5, 8, 31, 32, 33					X					
Every year there are more and more antibiotic-resistant infections. The problem of AMR will get worse over time. We need to take immediate action.	3, 6					X					
Antibiotic-resistant infections spread from one person to another person AND from animals to humans.	37, 45, 61, 68, 69					X					
Antibiotic-resistant infections are harder to treat than infections that are not resistant to antibiotics.	5, 6					X					
There are many simple behaviours we can do to make AMR less likely to happen, and keep ourselves, our families, communities and animals healthy and productive.	37–44 inclusive					X					
Using the wrong medicine, in the wrong amount or for the wrong amount of time could cause AMR.	7, 11, 21						X	X	X	X	
Symptoms need to be checked by a professional. It’s not always obvious whether an infection is viral or bacterial.	10, 17		X	X			X		X		
Care for a sick person by providing good easily digested food in small quantities, reducing fever by using warm bathing or fanning and giving anti-inflammatories increasing fluid intake enabling rest.	42						X				
By preventing infection and unnecessary antibiotic use we are preventing AMR from developing.	37–44 inclusive, 61–65 inclusive							X			
You can minimize unnecessary antibiotic use and AMR by keeping yourselves, your family, your animals and fish healthy.	48–60 inclusive							X			
Vaccinations prevent both humans and animals from getting sick from some infectious diseases.	45, 49							X			
Studies have found that washing your hands with soap and water at key times can greatly reduce the risk of infections.	63							X			
To prevent infections, regularly wash hands thoroughly with soap or alternative before eating or preparing food or dealing with infant faeces, and after contact with faeces (human and animal), after handling animals.	63							X			
All infections, including resistant infections, can be spread between people, animals and the environment.	37, 45, 61, 68, 69								X		
Faeces, food products, dirty water and contaminated soil (soil that has human and animal waste, or other types of pollution washed into it in large quantities) are some of the ways infections, and resistant infections, can move between people, animals and the environment.	54, 60, 74, 77								X		
Only give antibiotics to animals and fish under veterinary supervision. Only use this medication if it has been prescribed by a licensed/registered veterinarian (or if unavailable call vet, or use paravet).	47–52, 56, 57								X		
Do not use antibiotics for growth promotion or to prevent diseases in healthy animals or fish. Instead provide animals and fish with fresh, healthy and nutritious food rather than adding antibiotics to their food and give newborn animals the colostrum from their mothers.	47–52, 56, 57								X		
Meat or other produce (e.g. milk) from an animal that has recently been treated with an antibiotic can contain antibiotics.	54, 60, 74								X		
Maintain the antibiotic withdrawal time and do not sell or slaughter your animal until the withdrawal period is over. [Ask your vet when to sell or slaughter because the withdrawal period varies depending on the antibiotic group and the type of animal and animal product (e.g. meat/milk).]	54, 60, 74								X		
Preventing cross-contamination between people, animals and the environment is an important way to stop infections and AMR spreading.	37–44 inclusive								X	X	
Ensure animals and fish are kept in areas where they are not crowded; this helps to minimize infections spreading and the need for antibiotics.	37–44 inclusive, 48–60 inclusive									X	
Build separate animal houses to reduce the risk of zoonotic infection or AMR spreading.	37–44 inclusive, 48–60 inclusive									X	

## Ethics

Ethical approval for the entire COSTAR project was granted by the University of Leeds Faculty of Medicine and Health ethics board in March 2020, case reference: MREC 20-034. In-country ethical approval was granted by The Nepal Ethical Research Council (Reference number 3098) and The Bangladesh Medical Research Council.

## Results

### Worked examples of the framework

Here we present worked examples of the framework in action for the COSTAR project. Because the same CDA intervention was being developed across two different contexts there are similarities within steps 1, 2, 4, 5, 7 and 8. However, steps 3 and 6—which focus on context—have substantial differences in terms of the community-produced data and contextual information utilized. These differences then impact steps 9 and 10, and thus we provide a generic overview of the framework in action plus case study evidence specifically around steps 3 and 6 in each of our delivery settings.

### Discussion

We present a 10-step framework to combine academic, contextual and modality-specific information to create meaningful *key content* for CE interventions that address One Health challenges. The framework allows logistical and setting-specific flexibility but is underpinned by academic rigour and contextual relevance. This ensures *key content* and resulting behavioural objectives address the focal problem in a meaningful way for the intended community, whilst limiting unintended consequences, most importantly harm.

Through a detailed case study, we have demonstrated this framework in action to develop a suite of behavioural objectives for two CDA interventions addressing the challenge of AMR in Nepal and Bangladesh. These key messages have been utilized to create the full complement of materials required for a CDA project including a visual flip chart and discussion guide, which allows non-specialist facilitators to deliver 8–11 community dialogue sessions to their community peers.^45^ During dialogues, communities will explore issues around AMR, identify which issues are important in their own context and decide on meaningful and sustainable action plans they can implement to minimize AMR. Without clear and contextual *key content*, the process of delivering the CDA would be extremely challenging to implement, particularly around the issue of AMR, which is not routinely discussed at community level in either Nepal or Bangladesh.^31,32,50–52^

The framework specifically helped the authorship team to balance different sources of knowledge within the content development phase. For example, AMR guidance at national and global levels frequently stipulate that one should seek advice from a qualified healthcare professional before using antimicrobial medicines, complete the course and return if they are not well again. However, in both our contexts, identifying a qualified professional, for example, is difficult due to lack of obvious credentials, such as a fixed place of work, and fraught with equity issues around access and affordability. COSTAR’s formative work showed that qualified providers such as community health care providers (Bangladesh) and community health volunteers (Nepal) may not always have different drug options or the right amount of a single drug to provide a full course, thus the patient may need to return multiple times. This is a complex issue to tackle yet it hinges on building trust with recognized health professions. Here our framework allowed us to weigh up the inadequacy of academic literature for our specific contexts with the feasibility of engaging in such active discussions with our focal community. *Key content* focuses on identifying qualified healthcare providers, trusting their capacity and knowledge, and then adhering to prescription practices and dosage guidance.

Similarly, the framework allowed authors to reflect upon the contextual appropriateness of emerging data on AMR. In this case we refer to the growing academic evidence around AMR and antimicrobial contamination in the environment. Although resistant microbes and genes are commonly found in soil and water, studies cannot always determine causation and directionality of transmission. Equally, in terms of contamination it is very difficult to distil the risks of antimicrobial run-off from farms, and decomposition of medical antimicrobials, from when medicines are disposed of inappropriately, into simple key messages. This is because so many variables such as temperature, soil composition, type of antimicrobial agent etc. impact the risks. Finally, many environmental studies are laboratory based, and very few have been conducted within the COSTAR settings of Comilla District, Bangladesh and the Kapilvastu region of Nepal. Hence there is a lack of clear, decisive academic evidence to support the development of environmental AMR objectives. However, formative work at the community level suggested that in COSTAR’s context people were concerned about the health of their environments and thus the framework allowed the incorporation of *key content* around, firstly, microbes in the environment and secondly the propensity for antimicrobial drugs to reach the environment via contaminated water. This allowed COSTAR to link content points around practising good home hygiene and animal husbandry with environmental AMR stewardship (as detailed in Table 4). As such, community members can be supported to engage in best practices that promote One Health in a variety of ways rather than focusing on the specific academic evidence around AMR in the environment.

A limitation of this framework is that it requires a significant investment of resources to implement successfully.^5,8,53,54^ For the One Health challenge of AMR, both external experts and local stakeholders across animal, environmental and human health areas are required to engage with and adapt the key messages at various stages, costing both time and money. However, whilst CE and broader co-design processes are more resource intensive than top-down alternatives such as expert consultations, research demonstrates that top-down approaches do not produce sustainable results. Thus, although cheaper, there is no value for money if they are unsuccessful.^55^ This issue has been repeatedly identified by staff working on other environmental health and development. For example, in 96 interviews with water and sanitation professionals working on the frontline of implementation in Malawi, South Africa, Tanzania and Zimbabwe, participants in 66 (69%) interviews identified a major cause of failure as the inadequate engagement of intended users/learners in determining their needs and incorporating these into programme designs (Barrington *et al.*, unpublished data). Another study, in India, investigated 20 community-based sanitation systems using fuzzy-set qualitative comparative analysis to determine the causes of success and failure. They found that almost all systems considered to have failed had not incorporated community participation in planning or the priorities of intended users.^56^ Thus, CE approaches can be more successful in achieving the outcomes most desired by those intended to benefit from the programming (i.e. intended users/learners).

Our case study exemplifies the importance of detailed formative work engaging community members through a range of activities, from household surveys to more involved projects such as the participatory video (PV) approach or a pilot CDA study. Formative steps have been integral to contextualize the final behavioural objectives and content of our long-term CDA study (Figure 1). As such, CE can be resource heavy at the point of co-development. However, for many research groups, pilot or formative research will be a key component of intervention development and thus can feed into this framework in a logical manner.^10,12,34^ Additionally, once created, the *key content* can be adapted at minimal cost via engagement with existing team members, wider stakeholders and the communities themselves. For example, communities in the current CDA project have been supported to provide feedback throughout the CDA process, which includes commentary on the information delivered, its appropriateness and clarity.^5^

This framework’s potential is not limited exclusively to the CDA modality of CE. It could be applied to guide the development of many other CE interventions, which require some content to be prepared in advance of delivery. The key features of the framework are that it allows multiple sources of knowledge around the focal problem to be shared with, and contextualized by and for, the focal community, and the chosen CE method. Similarly, it could be applied to a range of One Health challenges beyond AMR; for example, zoonotic and vector-borne diseases, neglected tropical diseases including snake bites, and climate health-related issues such as air and water pollution, and adaptation to temperature change. Again, the key feature of the framework is the combination of academic and contextual information around the focal problem, which is then adapted to the proposed method of delivery.

In conclusion, we hope this framework will support the development of meaningful and contextually appropriate CE interventions that address a range of One Health challenges.

## Supplementary Material

dlad124_Supplementary_Data

## Supplementary data

The Case study and Tables S1 and S2 are available as Supplementary data at *JAC-AMR* Online.
