# Supplementary material for: Addressing antimicrobial resistance through community engagement: a framework for developing contextually relevant and impactful behaviour change interventions
Source: JAC Antimicrob Resist. 2023 Nov 24;5(6):dlad124. doi: 10.1093/jacamr/dlad124 (PMC10673675; doi:10.1093/jacamr/dlad124)
Supplement: dlad124_Supplementary_Data [file dlad124_supplementary_data.zip › Singular Framework Case Study .docx]

1. Thematic areas for *Key Content* were drawn from World Health Organisation (WHO) guidance on tackling AMR. This freely available guidance includes specific points for individuals, policymakers, health care professionals and the agricultural sector; globally it is considered the most recognisable, succinct, and accessible guidance on tackling AMR. COSTAR prioritised the individual and agricultural guidance which together comprises eleven statements on optimal antimicrobial use and infection prevention in humans and animals.
2. To ensure *Key Content* captured the One Health dynamics of AMR, COSTAR sought to broaden the list of content points by considering broader guidance on our topic of interest. This included discipline-specific documents from across the quadripartite alliance on AMR. This includes the Food and Agriculture organisation (FAO), World Animal Health Organisation (formerly OIE, now WOAH) and the United Nations Environmental Program (UNEP). The team also considered the National Action plan on AMR for the focal country plus the WHO’s Global AMR Action Plan, to ensure COSTAR’s *Key Content* reflected the objectives listed within these documents (Table S1).
3. The depth of *Key Content* was developed by considering community-level knowledge on AMR. This differed for each country setting as the COSTAR team utilised bespoke community engagement solutions to encourage community input and consolidate community knowledge. These included rapid qualitative studies, participatory video projects, household surveys etc. (For worked examples see Table S2).
   1. For example, in Bangladesh this included reviewing findings of a 2018 household survey on community level knowledge, attitudes, practices, and languages around the challenge of AMR, and the feedback and process evaluation data from the pilot iteration of the CDA approach in 2018. Specific contextual changes were made based on this, for example, K*ey Content* was added around the appropriate use of antimicrobials in fish farming which is a common form of agriculture in this area of Bangladesh but is not considered in detail by WHO AMR guidance.
   2. In Nepal the team conducted a household survey, a preliminary market assessment of antimicrobial use and sales in the delivery region, and a participatory video (PV) intervention allowing community members to share their stories of AMR through short films. Stories created during the PV process were used to contextualise K*ey Content* points which focused on the message that freely available antimicrobials from health posts are of high quality and efficacy. It is a popular myth in Nepal that costly drug-store antimicrobials are more effective than government supported free medicines. The PV storyline created by community members dispels this myth via relatable family health examples.
4. Finally, the team added their own subject-specific knowledge. COSTAR is comprised of a diverse international and interdisciplinary team. An open consultation with all team members allowed them to add to the existing list of *Key Content* and suggest relevant literature to support, shape and exemplify each content point. In COSTAR this was achieved by interactive online discussions using breakout rooms in software such as Zoom and Microsoft Teams. The team includes 35 core members based in 4 countries representing the human, animal, and environmental health dimensions of AMR, including water, sanitation, and hygiene behaviours and infrastructure.
5. Based on these steps, we created a list of 78 content points (Table 2) to minimise AMR at the community level. Many were the same for both settings whilst some required re-framing based on contextual information. As discussed, Bangladesh key messages included details on fish farming practices and fish health, which were not as relevant for the Nepali setting. In Nepal the focus was on poultry and buffalo animal husbandry. However, 77 content points are clearly too many to share within a series of 45-minute Community Dialogue sessions at community level. The team now wished to concentrate this list based on which objectives were most relevant to the focal community, had enough evidence to support simple behavioural actions and were possible to share within 45-minute sessions led by volunteer community facilitators.

1. The first step in refining the content list was to apply a contextual lens by asking; how much of the *Key Content* is relevant within our focal community? This lens was applied by engaging with wider stakeholders in each country who reviewed materials at several points during their development. Stakeholders included policy makers, local government officials and independent consultants within human or animal health, environment, and agriculture.

A commonality between the contextual refinements made in each setting was that *Key Content* needed to clearly differentiate between antimicrobials and other medicines. This led to a strong focus on disease-causing microbes, symptoms of infections and the need to seek health professionals’ advice before treatment. However, other applications of this step were different within each setting. In Bangladesh the 2018 pilot data was utilised whilst in Nepal the COSTAR team referred the two rounds of Participatory Video (PV) which were completed in 2022, see step 3. These applications allowed the COSTAR team to determine which *Key Messages* are most critical within each setting and what level of expansion is needed.

- 1. For example, in Bangladesh Community Health Care Providers (CHCPs) are well-respected and established figures in the community who can prescribe some antibiotics and antifungals. Thus, *Key Messages* which direct communities to a qualified health professional in Bangladesh can be quite simple.
  2. Alternatively, in Nepal there is some reluctance to visit community health posts where free antimicrobials are available because of the perception that these medicines are of lower quality. Thus, our messaging around health professionals, and particularly free medication, had to be presented differently in each context.

1. The second lens applied was to consider the strength of the academic evidence from *Step 4*. For example, academic evidence shows that trace antimicrobials can be present in the meat, milk and egg products of animals undergoing antimicrobial treatment. As such, consumption of such products should be halted until all traces of the antimicrobial have passed through the animal’s system, known as the withdrawal period. A suitable *Key Content* point thus could be to suggest people refrain from eating these products whilst an animal is on treatment and until the withdrawal period is over. However, in practice this is very difficult to communicate for several reasons. Firstly, in rural communities, animal proteins are a crucial part of a healthy diet. Thus, discussing contamination of animal-based products could cause harm in terms of reduced nutrition or a reluctance to use antimicrobials on sick animals. Secondly, the academic evidence regarding how long a withdrawal period should be is fraught with technicalities around the type of antimicrobial used, the dosage and the animal it is being used on. This makes it challenging to communicate a simple key message around how long one should wait to consume products form a sick animal. Equally a message around seeking advice from a health care professional may not be appropriate in our rural communities where animal health practitioners are unlikely to have detailed information regarding withdrawal periods.  Expecting community level animal health experts to be able to provide such detailed guidance, when the literature cannot, appears an unrealistic expectation and could actively cause harm within a community if people begin to distrust animal health providers.
2. The final lens applied was the fit of this *Key Content* to the Community Dialogue Approach itself. The CDA is a participatory approach supported by community-based volunteers. It is not a top-down information-giving session and requires active engagement from the focal community to develop enquiry and solutions to the focal challenges. As such the *Key Content* provided to guide the dialogue and train the facilitation team must be easily digested, relatable and simple to communicate at community level. For this reason, not all the *Key Content,* despite being contextually appropriate and having a sound basis of academic evidence, could be included in the final materials. For example, mitigating the risk of infections and trace antimicrobials spreading through the community’s wider environment (soil and water) was captured within in *Key Content*. However, in both settings the COSTAR team and wider stakeholders felt that this concept was considered very challenging to communicate in 45-minute dialogue sessions led by volunteer facilitators. As such, *Key Content* focused on keeping the environment clean, returning left over antimicrobials to health professionals where possible, and ensuring faecal matter does not contaminate the drinking water of humans or animals.
3. At this point the whole COSTAR team reviewed the *Key Content* and identified areas of overlap. This particularly related to the One Health focus of COSTAR. For example, in both childcare and animal husbandry, a key message was to vaccinate animals and children to prevent infection and later use of antibiotics thus reducing the risk of AMR developing. Rather than include these as two separate key messages 1) vaccinate children and 2) vaccinate animals. These were combined into one Key Message: To prevent infection and the development of AMR ensure all children, livestock and domesticated animals receive their vaccinations. In both our contexts this was an appropriate message to give as government- funded vaccination programmes are in place for common childhood and livestock diseases, and rabies for domesticated and feral dogs. This process ensured *Key Content* captured the need to take a One Health approach to the challenge of AMR and seeing human-animal-environmental health issues as one singular problem. This also helped shorten the list of *Key Content* and thus consolidate the content and material development process.
4. Steps 6-8 were repeated twice in both the Nepal and Bangladesh content development process. The second iteration of the steps was during the Training of Master Trainers, where a selection of local stakeholders such as policy makers, vets, pharmacists, doctors, and environmental scientists were trained on the CDA approach and materials. These individuals were asked to review in detail the materials used in the CDA and had the opportunity to re-shape and question the Key Content based on their contextual and academic understandings of AMR. This allowed the COSTAR team to make use of new academic evidence and community knowledge as it emerged in real time. The process resulted in a final list of:
   1. Nepal: 34 key content points across eight Community Dialogue sessions see Table 3
   2. Bangladesh:39 key content points across eight Community Dialogue sessions see Table 4
