# Supplementary material for: Addressing antimicrobial resistance through community engagement: a framework for developing contextually relevant and impactful behaviour change interventions
Source: JAC Antimicrob Resist. 2023 Nov 24;5(6):dlad124. doi: 10.1093/jacamr/dlad124 (PMC10673675; doi:10.1093/jacamr/dlad124)
Supplement: dlad124_Supplementary_Data [file dlad124_supplementary_data.zip › Supplementary files.docx]

Table S1

| **Resource** | **Publisher** | **Link** | **Details** | **Benefits** | **Limitations** |
| --- | --- | --- | --- | --- | --- |
| **World Health Organisation (WHO), Fact Sheet on Antimicrobial Resistance** | | <https://www.who.int/news-room/fact-sheets/detail/antibiotic-resistance>  Scroll down to *prevention and control* section | **Starting point for this intervention.**  Globally recognized document regarding action on AMR. | Clear list of behavioral objectives to minimize the development and spread of AMR.  Simple language.  Good consideration of Water, sanitation and hygiene, food, agriculture, and health behaviors. | Primary focus human health, although agriculture details added later, no environmental AMR information.  Not community centered. Language is directed toward professionals and policy makers. |
| **Quadripartite alliance on AMR’s strategic framework** | Food and Agriculture Organization (FAO) | Download the report here: <https://www.fao.org/documents/card/en/c/cb8766en> and general information here: <https://www.fao.org/antimicrobial-resistance/quadripartite/who-we-are/en/> | World Health Organisation (WHO), Food and agriculture organization of united nations (FAO), World Organization for Animal Health (WOAH) and the United Nations Environmental Programme (UNEP) make up this cross-cutting group. | Aim to preserve antimicrobial efficacy and ensure sustainable and equitable access to antimicrobials for responsible and prudent use in human, animal, and plant health, contributing to achieving the Sustainable Development Goals (SDGs).  Takes holistic One Health approach. | Policy-centered language, not geared toward community action. |
| **Food and Agriculture Organization (FAO) guidance on antimicrobial resistance** | | <https://www.fao.org/antimicrobial-resistance/resources/publications-archive/en/>  Specifically: <https://www.fao.org/documents/card/en/c/cc3856en> and <https://www.fao.org/documents/card/en/c/cc0927en> | | Extensive detail regarding AMR in agriculture (especially livestock) with reports providing specific details for different sectors (policy makers, farmers, environment, food chain) | Not community centered. Language is directed toward professionals and policy makers. |
| **World Organization for Animal Health (WOAH) guidance on antimicrobial resistance** | | <https://www.woah.org/en/what-we-do/global-initiatives/antimicrobial-resistance/> (bottom of page) and <https://www.woah.org/en/what-we-do/global-initiatives/antimicrobial-resistance/#ui-id-4> | Comprehensive webpages regarding the drivers and impacts on AMR in animal health. | Summary information regarding AMR in animal health. Sector specific information for farmers, vets and the lay person.  Engaging graphics and simple language.  Community-centered action points at bottom of page | Not as much detail as similar reports but more engaging and accessible. |
| **Resource** | **Publisher** | **Link** | **Details** | **Benefits** | **Limitations** |
| A summary for policy makers: The environmental dimensions of AMR. | **United Nations Environmental Programme (UNEP)** | <https://www.unep.org/resources/report/summary-policymakers-environmental-dimensions-antimicrobial-resistance> | Summary report calling for the strengthening of environmental action within the ‘One Health’ response to AMR. | Report discusses recent data on pollutants that exacerbate AMR in the environment, where such pollutants originate and provides an overview of the environmental action required to tackle AMR. | Policy level document with complex language  Not community centered. Language is directed toward professionals and policy makers. |
| Initiatives for Addressing Antimicrobial Resistance in the Environment: Current Situation and Challenges | **Wellcome Trust** | <https://wellcome.org/sites/default/files/antimicrobial-resistance-environment-report.pdf> | Summary of current knowledge and risks of AMR in the environment prepared by UK-based funder, The Wellcome Trust. Actions are geared toward policy makers, funders, and professionals. | Clear but detailed report regarding the drivers and impacts of AMR in the environment with suggested technical actions for policy makers, pharmaceutical companies (and other polluting agencies) and farmers/agricultural sector. | Policy level document with complex language  Not community centered. Language is directed toward professionals and policy makers. |
| No Time to Wait. Securing the world from Drug Resistant Infections | **Interagency coordination group (IACG) on AMR** | <https://www.who.int/docs/default-source/documents/no-time-to-wait-securing-the-future-from-drug-resistant-infections-en.pdf?sfvrsn=5b424d7_6>  Key messages on page 3 | Based on the UK government’s O'Neill report of 2016 this paper summarizes current knowledge of the risks and drivers of AMR with predictions for the future challenges, costs, and death tolls. Suggested actions for policy makers. | Very detailed report with strong focus on all aspects of AMR (not just bacterial AMR) focus is also cross cutting including human and livestock health, and the environment. | Policy level document with complex language  Not community centered. Language is directed toward professionals and policy makers. |
| **Nepal National Action Plan on AMR** | Government of Nepal | <https://cdn.who.int/media/docs/default-source/antimicrobial-resistance/amr-spc-npm/nap-library/national-antimicrobial-resistance-containment-action-plan_nepal.pdf?sfvrsn=367a5d0e_1&download=true> | National document created with support from WHO template for National AMR Action Plans | Provides assessment of AMR-related challenges in Nepal although much is unknown due to lack of surveillance and reporting mechanisms. Sets out ambitious plan for change over the next five years with clear actions to reduce and track AMR more effectively. | Detailed governmental document – not community centered although does discuss the need to engage the public with AMR more effectively.  Focus on human health. |
| **Resource** | **Publisher** | **Link** | **Details** | **Benefits** | **Limitations** |
| **Bangladesh National Action plan on AMR** | Bangladesh Government | <https://cdn.who.int/media/docs/default-source/antimicrobial-resistance/amr-spc-npm/nap-library/antimicrobial-resistance-containment-in-bangladesh-2017-2022.pdf?sfvrsn=bfa46b_3&download=true> | National document created with support from WHO template for National AMR Action Plans | Overviews the current AMR situation in Bangladesh across One Health sectors and outlines objectives to improve surveillance and diagnostics as well and antimicrobial stewardship, training and prescribing practices in human and animal health. | Detailed governmental document – not community centered although does discuss the need to engage the public with AMR more effectively.  Moving into One Health but still biased toward human health sectors. |
| 5-year plan from the UK government to reduce AMR | **UK Government** | <https://assets.publishing.service.gov.uk/government/uploads/system/uploads/attachment_data/file/1070263/UK_AMR_5_year_national_action_plan.pdf> | 5-year plan from the UK government to reduce AMR | Vast list which approaches the problem of AMR with a long-term perspective.  Makes clear the problem; its wide-ranging One Health impact and the many areas where changes will be required (unlike other country specific guidance this covers environmental and animal issues). | Useful for policymakers.  Does not give actions for individual members of the public.  May be inaccessible to someone with little to no knowledge of this issue. |
| TARGET antibiotics toolkit | **Royal College of General Practitioners (UK)** | <https://elearning.rcgp.org.uk/course/view.php?id=553> | Guidelines and principles for GPs for safe prescribing antibiotics | Contains information accessible to healthcare professionals and members of the public (see “Leaflets to share with patients”) | Approaches from a purely healthcare perspective. |
| The Core Elements of Hospital Antibiotic Stewardship Programs: 2019 | **US Center for disease control** | <https://www.cdc.gov/antibiotic-use/healthcare/pdfs/hospital-core-elements-H.pdf> | Guidelines and assessment criteria to evaluate whether hospitals in the United States responsibly used antibiotics | Give specific interventions that can be done in a secondary healthcare setting (i.e., education, reporting antibiotic use). The assessment criteria are also useful to provide unbiases assessments of antimicrobial stewardship. | Not accessible to those not in secondary healthcare setting.  Not community based.  Aimed at policymakers and healthcare professionals: layperson will have limited interaction with this. |
| **Resource** | **Publisher** | **Link** | **Details** | **Benefits** | **Limitations** |
| Antimicrobial stewardship: systems and process for effective antimicrobial medicine use | **National Institute for Health and Care Excellence (NICE), UK** | <https://www.nice.org.uk/guidance/ng15/chapter/1-recommendations> | Report reviewing how NICE recommendations are being implemented with respect to antimicrobial resistance | Addresses core reasons why AMR can occur (e.g., looks at alternative funding models to incentivize the development of new antimicrobials and examines whether current guidelines on AMR use are being adhered to). | Highly specific to healthcare settings (doesn’t address other causes) |
| **Guideline recommendations and antimicrobial resistance: the need for a change** | BMJ open access | <https://bmjopen.bmj.com/content/bmjopen/7/7/e016264.full.pdf> | Peer reviewed the study to assess whether prescribing guidelines (globally) inadvertently contributed AMR as they did not account for resistance patterns. | Good as highlights the need for effective guidelines therefore a comprehensive public health response to deal with AMR. | Not applicable to a wider public, specific to those with public health background.  Specifically applies to those who make public health policy.  Does not address wider causes of AMR (environmental, livestock) |
| **Antibiotic Use, Its Resistance in Nepal and Recommendations for Action: A Situation Analysis** | NIH National Library of Medicine | <https://pubmed.ncbi.nlm.nih.gov/26744193/> | Peer reviewed article concerning the current state of antibiotic use in Nepal. | Provides detailed background on antibiotic use in the health care sector and some commentary regarding livestock and farming sectors. | Not applicable to a wider public, specific to those with a research background. |
| Situation analysis: antibiotic use and resistance in Nepal | **Global Antibiotic Resistance Partnership** | <https://onehealthtrust.org/wp-content/uploads/2017/08/garp-nepal_es.pdf> | Thorough overview of state of AMR in Nepal | focus on humans and food producing animals. | Limited environmental or surveillance discussions. Lack of knowledge and capacity for such analyses in country cited as the reason for this.  No clear objectives for action. |
| National AMR containment plan for Nepal | Nepal government and Flemming Fund | <https://www.flemingfund.org/wp-content/uploads/3d9bf4b7ab190c600921a99cf1803059.pdf> | Discusses Nepal’s current knowledge of and approach to tackling AMR | Many areas overlap with Nepal National AMR Action Plan (see above) | |
| **Resource** | **Publisher** | **Link** | **Details** | **Benefits** | **Limitations** |
| Antimicrobial resistance summary | **Food standards agency (UK)** | <https://www.food.gov.uk/business-guidance/antimicrobial-resistance-amr> | Guidance and practices for the public to reduce AMR | Useful as uses accessible language and the advice given can be taken by anyone: is not just about healthcare setting and highlights how food management (cooking and washing) can reduce AMR spread. Contains video for those with limited comprehension abilities. | Good introductory resource but lacks detail for professionals. |
| Fact sheet: Guidance on AMR in the dairy sector | **International dairy federation** | <https://fil-idf.org/wp-content/uploads/2017/05/Factsheet-003_2017-Guidance-on-Antimicrobial-Resistance-from-the-Dairy-Sector.pdf> | Guidance and overview of the use of antimicrobials in dairy farming | Introduces AMR and practical advice for farmers as well as outlines the role that other bodies and stakeholders can have (e.g., pharmaceutical companies).  Uses nonscientific language that should be accessible to those not of a clinical background. | Only specific to dairy farming- although most of the information could be applied to other livestock farming. |
| AMR fact sheet | Pan American health organization | <https://www.paho.org/en/topics/antimicrobial-resistance> | Introduction to the concept of AMR including its impact and mechanisms. | Does not assume the reader has any prior knowledge of the subject and uses clear language that is likely accessible to those not fluent in English and may easily be translated. Approaches the issue from multiple angles (as well as healthcare impacts the piece mentions spread between humans and animals). | Does not mention environmental spread and guidance is not in very much detail. Advice is so broad as to almost be useless. |
| **Resource** | **Publisher** | **Link** | **Details** | **Benefits** | **Limitations** |
| Antibiotic use in Bangladesh: Situation analysis and recommendations | **One Health Trust** | <https://onehealthtrust.org/publications/reports/antibiotic-use-and-resistance-in-bangladesh/> | Report covers current knowledge of AMR in Bangladesh and highlights areas of weakness and recommendations for action | Detailed and clear. Attempts a One Health focus but is compromised by lack of existing data. | Does not mention fisheries and aquaculture which are a key source of food in Bangladesh and sink for antimicrobials.  Language is geared toward professionals and policy makers. |
| AMR in Bangladesh’s aquaculture industry | **CGIAR antimicrobial resistance Hub** | <https://amr.cgiar.org/blog/one-health-perspective-antimicrobial-resistance-bangladeshs-aquaculture> | Blog detailing Bangladesh’s specific challenges with AMR through aquaculture | Engaging language and format with clear explanations of why AMR is such a threat to One Health and why antibiotic use in the fisheries sector/aquaculture industry is driving this.  Clear recommendations for policy makers. | Not community based.  Aimed at policymakers and healthcare professionals: layperson will have limited interaction with this. |
| **Antimicrobial Resistance Guidance by the US food and drug administration (FDA)** | | <https://www.fda.gov/animal-veterinary/guidance-industry/antimicrobial-resistance-guidances> | | Collection of guidelines from the FDA regarding the use of antimicrobials in animal agriculture to prevent the occurrence of AMR. | Highly specific and technical guidance that only deals in animal agriculture. |

Table S1: The wider range of Global Guidance documents regarding antimicrobial resistance that were assessed by COSTAR team members to create the *Key content* points for community-facing materials. Please note all web links were active as of June 2023.

Table S2

| **Suggested Key Content point** | **Contextual information sources: Nepal** | | | | **Discussion based on contextual information sources** | **Discussion based on wider information sources** |
| --- | --- | --- | --- | --- | --- | --- |
|  | **1** | **2** | **3** | **4** |  |  |
| Different microbes cause different illnesses, and these will often require different treatment. | X | X | X |  |  |  |
| Antimicrobials are lifesaving medicines but should only be used when needed in human and animal health. | X |  |  |  | Most of the population may not understand the term antimicrobial.    The term *Antibiotic* is commonly recognized but there are still people who are unsure of its definition. | Is lifesaving appropriate? Life enhancing? |
| Difference medicines are needed to treat different illnesses | X | X | X | X |  | Show some clear examples to differentiate antimicrobials from pain killers and long-term illness medicine such as for cancer or diabetes. |
| Ask your health care provider or vet for guidance on the medicines you have been prescribed. | X | X | X | X | The community often does not know what type of medicine they/their children/their animals have been prescribed. | Need a key message around advocacy. Asking health care provider about the medicine and how to take it This should include the time it may take to feel better and any expected mild side effects which should not stop you taking it as advised. |
| The cost of a medicine does not always determine its quality or strength, it is more important to get the right medicine to treat your illness and only a health care provided can determine this. | X | X | X |  | Antibiotics are often considered ‘strong’ medicines when purchased from pharmacies, but weak when coming from voluntary health care providers | Careful not to conflate this with the cost of AMR. AMR costs money because illness become more difficult to treat and require more visits to the health provider, changes in medicine/longer treatment times. Not simply more expensive or *better* medicine. |
| You should only take antimicrobial medicines after consulting with a trained health care professional. They will decide what type of medicine you need and advise you how much to take for how long.    This will ensure you/your child/animal gets better but also that the antimicrobial medicines keep working for longer. |  |  | X | X | This is likely to be much harder in animal health because of the limited access to agro vets in this area.    Anyone, even untrained personnel, can prescribe antimicrobials and they are easily available everywhere.    In practice, many people without proper registration or license, have been running their business in the sector of agriculture and animal farming. | Need to explain what a qualified health care working is/ what they look like. |
| **Suggested Key Content point** | **Contextual information sources: Nepal** | | | | **Discussion based on contextual information sources** | **Discussion based on wider information sources** |
|  | 1 | 2 | 3 | 4 |  |  |
| A qualified health care provider is… give visual examples | X | X | X |  | Traditional Healers can be the first port of call when someone is sick – especially if they are from a lower socio-economic background and/or located far away from a health post.    Distance and money are the most important factors that contribute to the misuse of antimicrobials – both in human and animal health. | Use community mapping exercise to identify nearest qualified health care provider and agro vets. |
| Always seek advice from a qualified health care professional if you think you need antimicrobials or other medicines. | X |  | X |  | People usually come to visit doctor’s clinic after taking antibiotics from local medical shops.    People will seek local advice for animal health issues before visiting an agro-vet and then finally visiting the veterinary hospital. |  |
| Do not demand antimicrobials if your health care provider says you do not need them. Instead listen and follow their advice on alternative treatments. | X |  | X |  | Highly educated persons may demand antibiotics or other antimicrobials. Likely because they are in a rush to get back to work when sick. | This message could be challenging considering that the market analysis shows medical centres will regularly sell just three days’ worth of medication at a time or Health Workers may not have a full course to provide and require people to return to complete the course. |
|  | X |  | X | X | Community health workers receive government provided antimicrobial stewardship training, however reluctance to prescribe antimicrobials can degrade trust and relationships with the community. |  |
|  | X | X | X | X | Health Workers may not have a full course to provide and require people to return to complete the course. |  |
| **Suggested Key Content point** | **Contextual information sources: Nepal** | | | | **Discussion based on contextual information sources** | **Discussion based on wider information sources** |
|  | 1 | 2 | 3 | 4 |  |  |
| AMR happens when microbes learn to resist the power of antimicrobial medicine. Our behaviours can help this happen more quickly | X | X |  | X |  | Need visuals to support this content point. |
| Always complete the full dose of antimicrobial medicines that you/your child/ animal has been prescribed.    Do this even if you feel better or your child/animal starts to act as though they are better.    Taking the full dose ensures all the microbe is killed and ensures you will not have to spend more money on more treatment later. | X | X | X | X | Medical doctors will counsel patients on completing doses of antimicrobials, however a patient’s financial circumstances can impact on the adherence to this advice.    Participatory Video participants correctly believed that non-compliance with dosage guidance could mean a disease will come back stronger however they were not concerned about the completion of the recommended dose for their animals. | The financial link of costs now versus in the future should be stressed here.    The One Health nature of AMR should be stressed here – consequences are the same for people and animals if dosage guidance is not followed |
| Do not store or share antimicrobials. Use only on the person or animal they have been prescribed to treat. | X |  | X | X | There are situations where people ask for a glut of antimicrobials to store or use on other persons who have not attended the appointment or on other animals in the future. |  |
|  |  | X |  |  | People in their community often used human medicines for treating domestic animals. |  |
| **Suggested Key Content point** | **Contextual information sources: Nepal** | | | | **Discussion based on contextual information sources** | **Discussion based on wider information sources** |
|  | 1 | 2 | 3 | 4 |  |  |
| Your health care provider will give you the exact amount of medicine you need to take in the right dosage for the right amount of time – there should be no ‘leftovers’ for human treatment. | X |  | X | x | Medical shops provide AB dose for 3 days, then after people come to visit the clinic if they felt unrecovered. | This message is quite challenging given the context revealed in the market analysis – particularly for animal health. |
|  | X |  | X | x | Agro-vets can be motivated by financial gain and will prescribe multiple types of antibiotics rather than the best option for the illness |  |
| Antibiotics do not treat viral infections. People and animals often get better from a viral infection on their own with rest, clean water and healthy food. More serious viral infections may need anti-viral treatment as prescribed by a health care provider. | X |  | X | x | For common respiratory infections, people visit medical centres to seek antibiotics and can be demanding. | Drug bug matching game could be used to stress this point. |
| Be patient with your health care provider.    They may not immediately prescribe medicines; they may wish to take blood/stool/urine samples from you/your animals. This helps the health care provide understand what is causing the illness and prescribe the best medicine. | X |  | X |  | Cultures for diagnosis are possible in some private human health care facilities but these take time for results to return | Limited scope for culture analysis in animals. There are only two labs with the capacity to conduct culture and antibiotic sensitivity tests (for government provided health care facilities) |
| Antimicrobials only treat infections they cannot prevent infections and they will not help health animals grow faster. To keep your animal most productive and protect antimicrobial drugs you should only use antimicrobials on sick animals. Healthy animals stay healthy and productive by being kept clean and well fed. | X | X |  | x | Antimicrobials are frequently used in poultry health for growth promotion and prophylaxis. | Many messages here to split up in content |
| **Suggested Key Content point** | **Contextual information sources: Nepal** | | | | **Discussion based on contextual information sources** | **Discussion based on wider information sources** |
|  | 1 | 2 | 3 | 4 |  |  |
| Do not throw waste antimicrobials into toilets/latrines/water sources instead return to a pharmacy or health care provider. | X |  | X |  | Typically, in Nepal medicines are disposed of anywhere, in the open field, drainage, pit, burned etc.    Some agro-vets, take back the remaining medicines | Need to explain WHY not to do this – use diagrams and visuals to show that active antimicrobials can pass through water into soil/crops/animals/other people and cause AMR to happen elsewhere. |
| Clean water should be prioritized for human and animal consumption.    Do not re-purpose water between groups of animals as this allows infection to spread.    Ideally use clean water to wash cooking utensils and animal feed stations | X |  |  |  |  |  |
| Good animal husbandry is key to minimizing infection and illness which keeps your animals healthy but also minimizes the likelihood of using antimicrobial medicines. | X |  |  |  |  |  |
| Do not allow untreated human faeces to reach the environment because this can allow microbes to spread between people and animals. |  | X | X |  | In rural areas, some communities still practice open defecation. This is most common in the older generations. |  |
| If an animal is sick or on antimicrobial treatment, keep its faeces separate to your usual manure pile and do not spread on fields for 6 weeks | X |  | X |  | Animal faeces are commonly stored in a pit before being used manure/fertilizer. |  |
| Vaccination is a very good way to keep you/your children/your animals healthy and protected from infection, it is a good investment and will save money in the long term.    Vaccination will also maximise productivity of your animals. | x |  | X |  | Some people keep records of vaccinations especially when in a specific vaccination card/document. | Need to emphasise the benefits of vaccination clearly and in both the short and long term. |
| If possible, avoid the consumption (or sale for consumption) of the meat/milk/eggs of sick animals and those on antimicrobial treatment (and for at least 1 week after sickness or drug use has ended) | x | X |  |  |  | Be very careful with this point as we do not want people to go hungry or risk under-nourishment. This is not just related to direct consumption but also sales of these products which brings income to the family. |

Table S2: A behind-the-scenes view at the development of *Key Content* points for the Nepal iteration of Community Dialogue materials. This table shows how the evidence for content was gathered from four sources: 1) 2018 pilot materials from Bangladesh, 2) Reflection notes from two rounds of participatory film-making with community members on the topic of AMR in the Kapilvastu District of Nepal 3)Preliminary Market assessment for antimicrobial usage in the Kapilvastu District of Nepal (publication pending, conducted January 2021), 4) A scoping review (currently under review) regarding the use of Community Engagement approaches to tackle AMR in Nepal, protocol can be accessed here: <https://doi.org/10.17605/OSF.IO/FV326> . Upper-case X demonstrates strong evidence for the content point, and the lower-case x weaker evidence or passing mentions.
